# 

**Published:** 1891-07-04

**Authors:** 


					H OMAS'S
WHICH HOSPLTAJ.
.Asaorr Western Infiama&y
WITH EXTRA NURSING SUPPLEMENT.
Satttbday,
July 4th, 1891.

				

